# Histological evaluation of the debris removal efficiency of activation of sodium hypochlorite solution at different concentrations

**DOI:** 10.1186/s12903-023-03244-z

**Published:** 2023-07-28

**Authors:** Esin Özlek, Eda Acikgoz, Nesibe Zeyneb Gökkaya, Ahmet Taşan, Fikret Altındağ

**Affiliations:** 1 Department of Endodontics, Faculty of Dentistry, The University of Van Yuzuncu Yil, Van, Turkey; 2 Department of Histology and Embryology, Faculty of Medicine, The University of Van Yuzuncu Yil, Van, Turkey

**Keywords:** Irrigation, Root canal preparation, Sodium hypochlorite, Irrigation activation technique, Er,Cs:YSGG laser

## Abstract

**Background:**

This study aims to histologically evaluate the efficiency of debris removal through activation of 2.5% and 5.25% NaOCI using laser, ultrasonic, and intracanal heating methods.

**Methods:**

Sixty-four maxillary central incisor teeth were randomly divided into two groups according to the irrigation solution (*n* = 32); 2.5% NaOCI and 5.25% NaOCI. Subsequently, the samples were further divided into four subgroups according to the final irrigation activation technique (*n* = 8); SubgroupA: Er,Cs:YSGG laser, SubgroupB: Ultrasonic, Subgroup C: Intracanal heating, Subgroup D: no activation. Generalized Linear Models and Bonferroni tests were used for statistical analysis (*p* < 0.05).

**Results:**

The effect of NaOCI concentration was statistically significant (*p* < 0.001). Furthermore, the activation of NaOCI by laser exhibited a statistically significant difference compared to the ultrasonic and intracanal heating methods (*p* < 0.001).

**Conclusion:**

The efficiency of root canal cleaning increases with higher NaOCI concentration. Activation of NaOCI also significantly enhances its effectiveness.

## Introduction

Sodium hypochlorite (NaOCI) is widely used as an irrigation solution in endodontics due to its favorable physicochemical and antibacterial properties [1, 2]. Although NaOCI effectively dissolves necrotic tissue and smear layer, its extrusion beyond the root canal into the periapical tissues can lead to cytotoxic effects [3]. Systemic allergic reactions, hemolysis, ulceration, and necrosis may ocur in living tissues. NaOCI concentrations ranging from 0.5% to 5.25% are commonly used in endodontic treatments [4]. Previous studies have shown that the tissue-dissolving and antimicrobial activities of NaOCI vary depending on the concentration, volume, and contact time of the solution, with higher concentrations demonstrating greater effectiveness. However, it is known that increasing concentration of NaOCI also increases its cytotoxic effects [5]. Consequently, recent research in endodontics has focused on enhancing the efficacy of low concentrations of NaOCI [6]. Studies have reported that activation NaOCI improves both smear layer removal efficiency and antibacterial activity [7].

Passive ultrasonic irrigation (PUI involves avtivating irrigation solutions used ultrasonic tips operating at frequencies of 25–40 kHz. This method increases the effective surface area of the solutions through ultrasonic vibrations generated in the root canal, leading to more efficient cleaning [8, 9]. Verna et al. demonstrated that both passive ultrasonic irrigation and laser activated irrigation enhanced the success of endodontic treatments compared to traditional methods [10]. Recent studies have indicated that intracanal heating of NaOCI enhances the effectiveness of the solution [11, 12]. However, it remains unclear whether intracanal heating of low concentrations of NaOCI can achieve sufficient cleaning compared to high concentrations.

Therefore, the aim of this study is to histologically evaluate the efficiency of pulp residue and debris removal using both 2.5% and 5.25% NaOCI solutions, in combination with activation through laser, ultrasonic, and intracanal heating methods during the final irrigation step.

## Materials methods

### Ethical approval

Ethical approval for this study was obtained from the Institutional Review Board and the Ethics Committee of the University (2020/09–07).

### Methodology

**Fig. 1:**
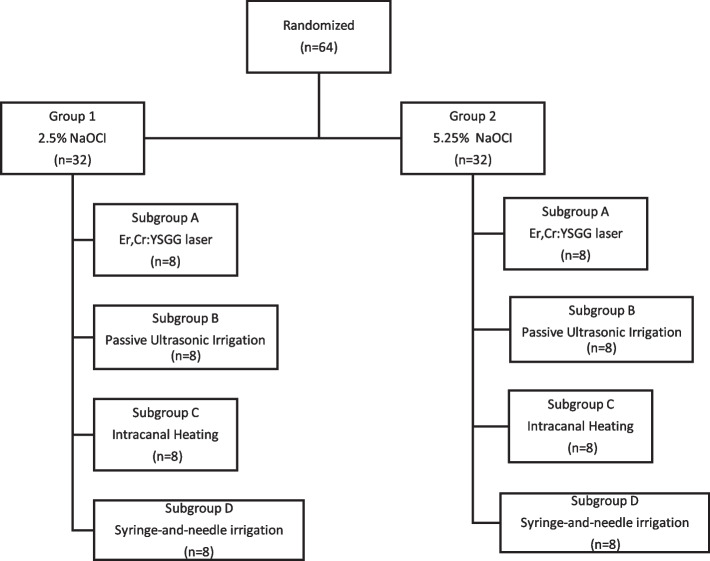
Distribution of groups

A total of 64 maxillary central incisor teeth with fully formed roots and closed apices were selected for his study. These teeth were extracted from individuals within the age group of 18–45 years for periodontal or orthodontic reasons. Prior to inclusion, the teeth were examined under a stereomicroscope, and any teeth with cracks or fractures were excluded. Only teeth without root canal calcification or resorption and with a minimum root length of 15 mm were included. Soft and hard tissue accumulations on the teeth were removed using a curette, and the teeth were stored in distilled water at room temperature until use. The teeth were decoronated using a slow-speed diamond saw with liquid cooling to obtain standardized root lengths of 15 mm. A size 10 K-type file was inserted into the root canal, and the working length was determined to be 1 mm shorter than the length visible at the apical foramen. The samples were randomly divided into two groups according to the concentration of NaOCI to be used during root canal instrumentation (*n* = 32); *G1; 2.5% of NaOCI* and *G2; 5.25% of NaOCI* (Fig. 1).

All samples were shaped up to X4 using the Protaper Next rotary file system(DenstplyMaillefer, Baillagues,Switzerland) and the X-Smart Plus (Dentsply Maillefer, Ballaigues, Switzerland) endodontic motor. Irrigation was conducted using 1 mL of either 2.5% or 5.25% NaOCI solution (Imicrly, Konya, Turkey) with a 31G side-vented needle during instrument change. Following instrumentation, the root canals were irrigated with 5 mL of 17% EDTA solution (ImidentTM Med., Konya, Turkey) for 1 min to eliminate the smear layer. No additional solution was utilized between the NaOCI irrigation and the subsequent EDTA irrigation. Afterwards, the samples were randomly divided into four subgroups based on the method of final irrigation activation (*n* = 8);

### Subgroup A: Er,Cr:YSGG laser

The root canals were filled with NaOCI solution and activated using Er, Cr: YSGG laser (Waterlase MD, Biolase Technology Inc., San Clemente, CA, USA) with an RFT2 tip. The tip, with a diameter of 275 microns in diameter and a length of 21 mm, was placed 1 mm short of the working length. The laser parameters were set to an output power of 2W, pulse frequency of 20 Hz, and a combination of 10% air and 10% water. The laser activation was performed in a slow and helical motion from the apical area to the coronal area for 8 s. The tip was positioned away from the dentinal walls and activated for 8 s, followed by a 10-s of deactivation period. This activation procedure was repeated 3 times, using fresh NaOCl solution for each cycle.

### Subgroup B: passive ultrasonic irrigation

NaOCI was activated using ultrasonic device (VDW, Munich, Germany). The device was set to a power setting of 30, and activation was performed using a 21 mm-IRR20 irrigation tip placed 1 mm behind the working length. The tip was positioned away from the dentinal walls and activated for 20 s. This activation procedure was repeated 3 times, using fresh NaOCl solution for each cycle.

### Subgroup C: intracanal heating

NaOCI solution was placed in the root canals and intracanal heating was performed using System-B device (11). System-B device was set to 150 ^0^C and an X-fine tip was placed in the root canal, positioned 3 mm shorter than the working length without touching the canal walls. The tip was positioned away from the dentinal walls and activated for 8 s, followed by a 10-s period of deactivation. This activation procedure was repeated 3 times, using fresh NaOCl solution for each cycle.

### Subgroup D (control): Syringe-and-needle irrigation

The root canals were irrigated with NaOCI solution using a 31-gauge closed-end needle (Ayset, Adana, Turkey), placed 1 mm short of the working length. The needle was moved in short vertical strokes with an amplitude of 2–3 mm at an approximate rate of 100 strokes per minute.

A total of 5 mL of NaOCI solution was used for irrigation in all samples. The activation process was performed for a total of 1 min with 3 cycles of 20 s each (8 s active, 12 s waiting). The solution was refreshed for each activation cycle. Following the activation process, all samples were washed with 5 mL of distilled water and dried with paper points.

### Histological evaluation

**Fig. 2:**
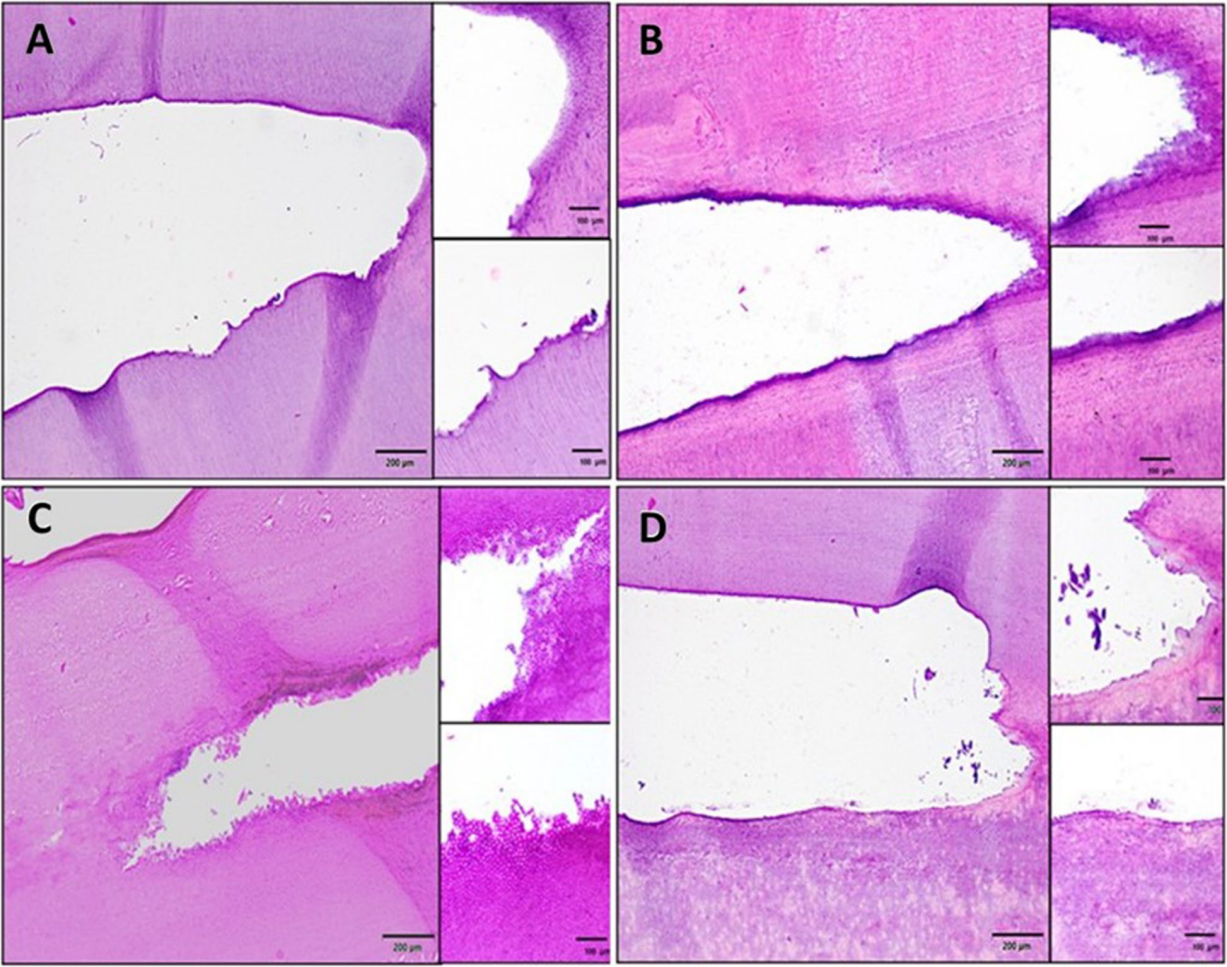
Representative histological picture from root canals section; (**A**) Er,Cs:YSGG laser, (**B**) PUI, (**C**) Intracanal heating, (**D**) no activation

After the completion of the endodontic procedures, all samples were fixed in 10% (v/v) neutral buffered formalin for 48 h and then washed under running tap water for 1 h. Decalcification of teeth was performed using a 5% nitric acid solution, with fresh solution being replaced every two days. The endpoint of decalcification was determined using a chemical method previously described in a study [13]. The tooth specimens were washed in running water for approximately 2 h and then dehydrated in ascending grades of ethanol, cleared in xylene, and embedded in paraffin. The samples were cut 5-μm-thick by rotary microtome (Leitz, Wetzlar, Germany) and stained with hematoxylin–eosin (Fig. 2). The sections were then viewed under a light microscopy at magnifications of 20X and 40X. Debris, including dentin chips, pulp remnants, and other particles loosely attached to the root canal walls, were defined as debris (K1). The images were placed on a grid using Image-J (Image J; National Institutes of Health, Besda, MD), and the percentage of remaining debris was calculated by two independent examiners [2, 14].

### Statistical analysis

The data were analyzed using IBM SPSS V23. The normal distribution of the data was assessed using the Kolmogorov-Smirnov test. The Generalized Linear Models method was used to evaluate the main effects of concentration and activation, as well as their interaction, on the amount of residual debris. Multiple comparisons were performed using the Bonferroni test. The results of the quantitative data analysis were presented as deviations. The level of significance was set at *p*<0.05.

## Results

The main effect of concentration on the amount of residual debris was found to be statistically significant (*p*<0.001) (Table 1). The average residual debris for the 5.25% contraction was 0.255, while it was 0.312 for the 2.5% contraction. There was no statistically significant interaction between the method and concentration on the amount of residual debris (*p*=0.713). The main effect of the irrigation activation technique on the amount of residual debris was statistically significant (*p*<0.001) (Table 2). The mean residual debris for laser activation was 0.143, for passive ultrasonic activation was 0.229, for intracanal heating was 0.320, and for the unactivated control group was 0.443. The control group had the highest mean amount of residual debris, while the laser group had the lowest mean amount of remaining debris.

**Table 1:** Descriptive statistics

Activation Technique					
Concentration	Er,Cs:YSGG laser	PUI	Intracanal heating	Control	Mean
%5.25	0.100 ± 0.072	0.206 ± 0.156	0.288 ± 0.162	0.425 ± 0.249	0.255 ± 0.208
%2.5	0.186 ± 0.125	0.251 ± 0.140	0.351 ± 0.189	0.461 ± 0.223	0.312 ± 0.201
Total	0.143 ± 0.110^a^	0.229 ± 0.149^b^	0.320 ± 0.178^c^	0.443 ± 0.236^d^	0.284 ± 0.206

^a^^−^^d^There is no difference between methods with the same letter

**Table 2:** Comparison of residual debris by activation technique and concentration

	Test statisticis^a^	Sd	*p*
Avctivation technique	185.388	3	< 0.001
Concentration	12.421	1	< 0.001
Activation technique ^a^ Concentration	1.369	3	0.713

^a^Wald Chi-square test statistic

Multiple comparisons revealed statistically significant differences in the mean amount of debris between the laser and passive ultrasonic activation, and between the laser and intracanal heating method, and between passive ultrasonic and intracanal heating method (*p*<0.001).

## Discussion

This study investigated the effects of different concentrations of NaOCI solution and various final irrigation techniques on the remaining pulp and debris in the root canal. The results showed that the 5.25% of NaOCI solution provided significantly better cleaning in the root canal compared to the 2.5% of NaOCI solution. Furthermore, when NaOCI solution was activated during the final irrigation, laser activation demonstrated a statistically significant difference compared to passive ultrasonic activation and intracanal heating method. Activation of the 2.5% of NaOCI solution using laser, passive ultrasonic, and intracanal heating method did not yield as much cleaning efficiency as the 5.25% of NaOCI solution.

Studies suggest that the toxicity of NaOCI increases with its concentration and that it should be used at the lowest effective concentration [5, 15]. Numerous studies in the literature have evaluated the effects of different NaOCI concentrations on antimicrobial activity, tissue dissolution, debris removal, and postoperative pain [15, 16]. However, despite these studies, there is still no consensus on the recommended optimal concentration of NaOCI. In a study by Siqueira et al. [17], which evaluated the antimicrobial effect of 1%, 2.5%, and 5.25% NaOCl, it was reported that all concentrations were effective, and the effect increased with higher concentrations. Ayhan et al. [18] compared the antimicrobial activity of 0.5% and 5.25% NaOCl on different microorganisms and found that the 0.5% concentration had a significantly lower effect. Baumgartner and Cuenin [19] reported that NaOCl concentrations of 0.5%, 1%, 2.5%, and 5.25% were capable of completely removing predentin and pulp tissue remnants from the unformed canal wall. In contrast, Goldsmith et al. [20] stated that diluting NaOCl up to 2.2% did not significantly reduce its tissue dissolving effect, but the effect of a 0.5% of NaOCl insufficient. More recently, Virdee et al. [6] reported that the penetration of the solution increased with higher NaOCI concentration and contact time. In the literature review, no study was found where the root canal cleaning efficiency of different NaOCI concentrations was evaluated histologically. Therefore, in this study, the debris removal efficiency of both 2.5% and 5.25% NaOCI solutions and different final irrigation techniques were evaluated. The results showed that the 5.25% NaOCI solution significantly improved root canal cleaning compared to the 2.5% NaOCI solution.

When reviewing the existing literature, a consensus emerges that as the concentration of the NaOCI solution increases, its effectiveness also increases. Consequently, 5.25% NaOCI solution is commonly preferred in clinical practice. Additionally, in recent years, irrigation activation methods have been recommended to enhance solution effectiveness. This raises the question, “Is it possible to achieve the effects of high concentrations of NaOCI by activating low concentrations of NaOCI solution? Does this reduce toxicity?'' In this study, 2.5% NaOCI solution was activated using different methods during the final irrigation, and the results were compared with those of 5.25% NaOCI solution. However, the findings indicate that the cleaning efficiency of the high concentration NaOCI solution could not be achieved by activating the low-concentration NaOCI solution using laser, ultrasonic, and intracanal heating methods during the final irrigation. Activation at both concentrations improved solution effectiveness, with Er,Cr:YSGG laser activation demonstrating more effective cleaning in the canal compared to PUI and intracanal heating methods. Notably, the difference between PUI and intracanal heating was significant. Wang et al. [21], Ozbay et al. [22], and Shahriari et al. [23] in their studies evaluating the smear removal efficiency of irrigation solutions with laser, reported that laser activation significantly enhanced solution effectiveness. Mancini et al. [24] also found that laser activation removed more smear layer compared to sonic and ultrasonic methods. Therefore, the results obtained in this study align with the existing literature.

In recent years, researchers have shown interest in increasing the efficiency of NaOCl through heating. Heating the NaOCI solution enhances its flow and reaction rate, resulting in increased antimicrobial activity and tissue dissolution effect[11, 25]. There are two methods of heating NaOCI in the root canal: extra-oral heating, where the solution is heated outside the canal, and intracanal heating, where heat carriers are used to heat the solution after it is placed in the canal. Iandolo et al. [11] reported that intracanal heating was more effective in their study comparing intracanal heating of NaOCI at 180 °C and extra-oral heating at 50 °C on root canal cleaning. Another study by Damade et. [25] compared the amount of debris removal using intracanal and extra-orally heated NaOCI solutions with and without ultrasonic activation. They found that intracanal heating removed more debris and had a significant effect. In contrast, this study found that ultrasonic activation cleaned more debris than intracanal heating. The conflicting results between these two studies may be due to differences in methodology.Damade et al. used heated NaOCI solution during root canal expansion and shaping, while it was used in final irrigation activation in this study. In another study by Landolo et al. [12], it was reported that ultrasonic activation of heated NaOCI increased the amount of debris removal compared to ultrasonic activation of non-heated NAOCI, showing a significant difference consistent with Damade et. al.’s results The boiling temperature of NaOCI solution ranges from 96 °C to 120 °C. In studies evaluating the effectiveness of NaOCI heating, researchers tested different temperatures. Woodmansey et al. [11] conducted their studies at 200 °C, Iandolo et al. [11, 12] at 150 °C, and Simeno et al. at 100 ^0^C. Iandolo et al. stated that heating NaOCI solution to 200 °C, which boils at 96–120 °C, would be meaningless, and repeating it in 3 cycles at 150 ^0^C would be sufficient. Simeone et al. [26] reported that heating the NaOCI solution up to 150 °C and repeating it in 5 cycles were sufficient, and this temperature was considered safe for the periodontal ligament. In this study, the temperature was set at 150 °C for 3 cycles based on Iandolo et al.’s study. While the results obtained from both the literatüre studies and this study are satisfactory, further studies, such as combining intracanal heating with ultrasonic activation, could potentially yield even better results [27].

NaOCI is commonly used as an irrigation solution in endodontic treatments due to its antibacterial activity and capacity to dissolve organic tissue. However, it does not have an effect on the inorganic components of the smear layer. Therefore, it is recommended to use proteolotic irrigants such as NaOCI in combination with chelating/calcifying agents like EDTA to remove the smear layer [28]. Two different irrigation protocols are used in endodontic clinical applications; *NaOCI-EDTA* and *NaOCI-EDTA-NaOCI *[12]. However, studies have reported that long-term use of EDTA as a final irrigation solution can lead to dentin erosion. In root canal treatments, NaOCI is used to dissolve organic tissues and for its strong antimicrobial activity, while EDTA is used to dissolve hard tissue residues and open dentinal tubules. Therefore, a final irrigation with NaOCI after EDTA is advantageous for disinfection of the opened dentinal tubules and prevention of dentin erosion caused by EDTA [2]. In this study, NaOCI was used during root canal shaping, followed by EDTA and NaOCI as the final irrigation, respectively. The aim was to evaluate the debris removal efficiency of activating NaOCI at different concentrations, as mentioned before. Only NaOCI, which was used as the final irrigation solution, was activated in this study. However, it should be noted that one limitation of this study is that only NaOCI was activated, and EDTA was not activated in the final irrigation.

Various methods are used to evaluate the cleanliness of root canals, including scanning electron microscope (SEM), optical microscope, computed tomography for pre/post instrumentation analysis, and histological analysis. However, none of these methods is considered ideal [29]. Although SEM is commonly preferred in the literature for the evaluation the smear layer in root canals, its use has been questioned in recent years because SEM images are two-dimensional and based on qualitative observation. Therefore, histological analysis has been used in conjunction with SEM to evaluate the amount of smear and debris in root canals, with the understanding that these two methods complement each other [30, 31]. In this study, only the amount of debris remaining in the root canals was evaluated, and the histological analysis, quantitatively assessed by two independent researchers, was chosen as the preferred method. Another limitation of this study is that the remaining smear layer on the inner walls of the root surface was not evaluated by SEM analysis.

## Conclusion

The 5.25% NaOCI solution provided significantly more effective cleaning of the root canal compared to the 2.5% NaOCI solution. Regardless of the final irrigation activation method used, it increased the effectiveness of the NaOCI solution at both concentrations. The Er,Cr:YSGG laser made a significant difference compared to passive ultrasonic irrigation and intracanal heating method. Ultrasonic activation provided more effective cleaning than intracanal heating and made a significant difference. However, it should be noted although activation of the 2.5% NaOCI solution with Er,Cr;YSGG laser, ultrasonic, and intracanal heating method in the final irrigation increased its effectiveness, it was not as effective as the 5.25% NaOCI solution.

## Data Availability

The datasets used and/or analysed during the current study available from the corresponding author on reasonable request.
